# Design, Synthesis of Novel Lipids as Chemical Permeation Enhancers and Development of Nanoparticle System for Transdermal Drug Delivery

**DOI:** 10.1371/journal.pone.0082581

**Published:** 2013-12-12

**Authors:** Srujan Marepally, Cedar H. A. Boakye, Punit P. Shah, Jagan Reddy Etukala, Adithi Vemuri, Mandip Singh

**Affiliations:** College of Pharmacy and Pharmaceutical Sciences, Florida A&M University, Tallahassee, Florida, United States of America; Louisiana State University Health Sciences center, United States of America

## Abstract

In the present study, we designed and developed novel lipids that include (*Z*)-1-(Octadec-9-en-1-yl)-pyrrolidine (Cy5T), 1, 1-Di-((*Z*)-octadec-9-en-1-yl)pyrrolidin-1-ium iodide (Cy5), (*Z*)-1-(Octadec-9-en-1-yl)-piperidine (Cy6T), and 1, 1-Di-((*Z*)-octadec-9-en-1-yl) piperidin-1-ium iodide (Cy6) to enhance the transdermal permeation of some selected drugs. Firstly, we evaluated the transdermal permeation efficacies of these lipids as chemical permeation enhancers in vehicle formulations for melatonin, ß-estradiol, caffeine, α-MSH, and spantide using franz diffusion cells. Among them Cy5 lipid was determined to be the most efficient by increasing the transdermal permeation of melatonin, ß-estradiol, caffeine, α-MSH, and spantide by 1.5 to 3.26-fold more at the epidermal layer and 1.3 to 2.5-fold more at the dermal layer, in comparison to either NMP or OA. Hence we developed a nanoparticle system (cy5 lipid ethanol drug nanoparticles) to evaluate any further improvement in the drug penetration. Cy5 lipid formed uniformly sized nanoparticles ranging from 150–200 nm depending on the type of drug. Further, Cy5 based nanoparticle system significantly (p<0.05) increased the permeation of all the drugs in comparison to the lipid solution and standard permeation enhancers. There were about 1.54 to 22-fold more of drug retained in the dermis for the Cy5 based nanoparticles compared to OA/NMP standard enhancers and 3.87 to 66.67-fold more than lipid solution. In addition, epifluorescent microscopic analysis in rhodamine-PE permeation studies confirmed the superior permeation enhancement of LEDs (detection of fluorescence up to skin depth of 340 μm) more than lipid solution, which revealed fluorescence up to skin depth of only 260 μm. In summary the present findings demonstrate that i) cationic lipid with 5 membered amine heterocyclic ring has higher permeating efficacy than the 6 membered amine hertocyclic ring. ii) The nanoparticle system prepared with Cy5 showed significant (p<0.05) increase in the permeation of the drugs than the control penetration enhancers, oleic acid and NMP.

## Introduction

Transdermal drug delivery offers several advantages over the conventional methods of drug administration such as avoiding the first pass metabolism, reducing systemic side effects and providing sustained release of drugs [1]. However, permeation of most of the drugs across the skin barrier remains a major challenge [2]. The upper layer of the skin, the stratum corneum (SC), impedes the flux of exogenous molecules across it and provides a strong barrier to permeation of most drugs into deeper dermal layers. The protective nature of the SC is attributed to its multilayered wall-like structure, in which terminally differentiated keratinocytes are embedded in an intercellular lipid-rich matrix. Various approaches have been developed in recent decades to overcome the skin barrier properties including physical approaches using iontophoresis, sonophoresis and microneedles [3,4], chemical approaches using penetration enhancers, and biochemical approaches using liposomal vesicles and enzyme inhibition [5,6,7,8].

Among the transdermal permeation enhancing approaches, the use of chemical penetration enhancers is the most suitable option owing to the low cost and ease of application [9,10,11]. These chemicals interact with constituents of the major skin barrier, stratum corneum, to promote drug ﬂux. Despite the fact that several compounds have been evaluated as permeation enhancers, their low permeabilities and skin irritation properties have limited their application in transdermal systems. Amphiphilic compounds such as azone, seven alkyl-6-(2,5-dioxopyrrolidin-1- yl) hexanoates and seven alkyl-6-(2,5-dioxopyrrolidin-1-yl)-2-(2-oxoazepan-1-yl)hexanoates, each containing a polar head and a lipophilic chain, are notable examples in this class of permeation enhancers. They each possess high activity coupled with low toxicity. These molecules are capable of inserting themselves into the stratum corneum, ceramide-rich lipid lamellae and disrupting their tight packing [12,13]. 

Among various skin permeating agents, the heterocyclic compounds such as azone and 6-amino hexanoates, are well-studied chemical penetration enhancers, which contain lipid alkyl chains and large polar head groups that have been determined to be vital for their respective activities [6,9]. This class of permeation enhancers has been shown to increase skin permeability by disordering or ‘fluidizing’ the lipid structure of the stratum corneum and forming micro cavities within the lipid bilayers, which ultimately increase the diffusion coefficient of a drug. In some cases, the enhancers penetrate into and mix homogeneously with the lipids. The activity of a penetration modifier as an enhancer or retardant is based on its molecular shape, H-bonding potential, polarity, chemical structure and the accompanying formulation. Interestingly, many Azone^®^-related analogs (including those containing modiﬁcations in the lipophilic chain and/or heterocyclic moiety) have been synthesized and examined as potential chemical penetration enhancers [14]. 

The naturally occurring unsaturated fatty acid, Oleic acid (OA) (18:1) is an FDA approved and widely used chemical permeation enhancer. OA interacts with and modifies the lipid domains of the SC [15,16]. Previous studies have suggested that OA stimulates lipid domains within the SC lipid bilayer thereby providing barrier defects within the lipid bilayer and thus facilitating the permeation of cargo molecules into the deeper epidermal layers (17, 18). Chong-Kook Kim et al., suggested that the pyrrolidone derivatives incorporated into liposomes increased the fluidity of the lipid bilayer in the liposome and such activity might have some correlation with the transdermal absorption-enhancing activity of therapeutic agents [17]. 

In the present study, we have designed and synthesized a new class of lipids containing heterocyclic head groups and oleyl hydrophobic chain domains. The compounds described in this investigation are novel and they exhibit high efficiency as transdermal penetration enhancers. Herein, the synthesis of a new series of chemical penetration enhancers is reported which includes (*Z*)-1-(Octadec-9-en-1-yl)-pyrrolidine (5 membered cyclic ring with tertiary amine, Cy5T), 1, 1-Di-((*Z*)-octadec-9-en-1-yl)pyrrolidin-1-ium iodide (5 membered cyclic ring with quarternary amine, Cy5), (*Z*)-1-(Octadec-9-en-1-yl)-piperidine (6 membered cyclic ring with tertiary amine, Cy6T), and 1, 1-Di-((*Z*)-octadec-9-en-1-yl) piperidin-1-ium iodide (6 membered cyclic ring with quarternary amine, Cy6). Firstly, we evaluated the transdermal permeation efficacies of these lipids as chemical permeation enhancers in vehicle formulations for drugs such as melatonin, ß-estradiol, caffeine, α-MSH, and spantide II using franz diffusion cells. We also used these lipids in the Lipid-Ethanol-Drug nanoparticles (LEDs) for improved permeation of these drugs. Additionally, we examined the retention of the LEDs in various skin depths using fluorescence microscopic analysis to demonstrate their high transmembrane permeability.

## Materials and Methods

### 1. Ethics statement

The animal protocol No.009-10 for the experiments was approved by Institutional Animal Care and Use Committee (IACUC) at Florida A&M University. All experiments were done in accordance with the guidelines of the Institutional Animal Care and Use Committee (IACUC) at Florida A&M University.

### 2. General procedures and reagents

^1^H NMR spectra were recorded on a Varian FT AV 300 MHz NMR Spectrometer. Pyrrolidine, Piperidine and Triphenyl phosphene, were purchased from Alfa Aesar (USA). Oleic acid, Iodine, Caffeine, ß-estradiol, Spantide-II and Melatonin were procured from Sigma-Aldrich (USA). α-MSH was procured from CHI Scientifics (USA). Polyoxyethylene-20 oleyl ether (Volpo-20) was a kind gift from Croda Inc. (Edison, NJ, USA). Phosphate buffer saline sachets (PBS, pH 7.4) were purchased from Sigma-Aldrich Co (St. Louis, MO, USA). HPLC grade of acetonitrile, water and ethanol were purchased from Sigma-Aldrich Co (St. Louis, MO, USA). N-methyl 2-pyrrolidone (NMP) was purchased from VWR International (Suwanee, GA, USA). Transcutol was a kind gift from Gettefossee (France). 

### 3. Syntheses

The synthetic route used for preparing the lipids 1-4 has been shown below.

#### Synthesis of 1-iodo-octadec-9-ene

In a round-bottom flask, PPh3 (25.2 mmol), imidazole (53 mmol), was dissolved in THF (30 mL) and iodine (23.6 mmol) was added at 4 °C. Oleyl alcohol (22.4 mmol) in THF (8 mL) was slowly added, and the reaction was maintained at room temperature for 24 h. THF was removed with a rotary evaporator, and the product was purified by flash chromatography using hexane. The product (intermediate I) was obtained as colorless oil with 79% yield.

^1^H NMR (300 MHz, CDCl_3_), δ 0.88 (t, J = 6.84 Hz, 3H), 1.17−1.44 (m, 22H), 1.71−1.86 (m, 2H), 1.97−2.05 (m, 4H), 3.19 (t, J =7.05 Hz, 2H), 5.29−5.41 (m, 2H);

#### Synthesis of (Z)-1-(Octadec-9-en-1-yl)-pyrrolidine (cy5T)

Intermediate I (500 mg, 1.32 mmol) prepared in step 1 above was dissolved in 50 mL of ethyl acetate in a 100 mL round-bottom ﬂask. Pyrrolidine (187mg, 2.64mmol) and K_2_CO_3_ (365 mg, 2.62 mmol) were added to the above solution. Reaction was stirred at 80°C for 24 h. The reaction mixture was diluted with 100 mL of ethyl acetate, and washed with water (2 x 100 mL). The organic layer was separated, dried over anhydrous sodium sulfate, and concentrated by rotary evaporator. The residue, upon column chromatographic puriﬁcation over 60-120 mesh size silica gel column using 4% methanol/chloroform (v/v) as eluent, afforded the pure title Lipid 1 as a sunset yellow liquid (361mg, 86.8% yield, Rf=0.3, in 5% methanol:chloroform).

^1^H NMR (300 MHz, CDCl_3_): δ 0.87 (t, 3H, -CH_3_, *J* = 7.0 Hz), 1.26-1.40 [(m, 24H, -(CH_2_)_12_-)], 1.62-1.72 [(m, 4H, -(CH_2_)_2_-], 2.14-2.20 [(m, 4H, -CH_2_-(CH=CH)-CH_2_-)], 2.46-2.58 [(m, 4H,-(CH_2_)_2_-*N*)], 3.45 (t, 2H, N-CH_2_, *J* = 7.8 Hz), 5.37-5.45 [(m, 2H, -CH_2_-(CH=CH)-CH_2_-)].

#### 1,1-Di-((Z)-octadec-9-en-1-yl) pyrrolidin-1-ium iodide (cy5)

Intermediate I (500 mg, 1.32 mmol) prepared in step 1 above was dissolved in 50 mL of ethyl acetate in a 100 mL round-bottom ﬂask. Pyrrolidine (46.2mg, 0.66mmol) and K_2_CO_3_ (91.25 mg, 0.66 mmol) were added to the above solution. Reaction was stirred at 80°C for 96 h. The reaction mixture was diluted with 100 mL of ethyl acetate, and washed with water (2 x 100 mL). The organic layer was separated, dried over anhydrous sodium sulfate, and concentrated by rotary evaporator. The residue, upon column chromatographic puriﬁcation over 60-120 mesh size silica gel column using 7% methanol/chloroform (v/v) as eluent, afforded the pure title Lipid 2 as a dark yellow semi solid (572mg, 66.4% yield, Rf=0.1, in 5% methanol:chloroform).

^1^H NMR (300 MHz, CDCl_3_): δ 0.86 (t, 6H, -CH_3_, *J* = 7.2 Hz), 1.26-1.42 [(m, 44H, -(CH_2_)_12_-)], 1.66-1.78 [(m, 8H, -(CH_2_)_2_-], 2.17-2.21 [(m, 8H, -CH_2_-(CH=CH)-CH_2_-)], 3.20-3.64 [(m, 8H,-(CH_2_)_4_-*N*)], 5.32-5.44 [(m, 4H, -CH_2_-(CH=CH)-CH_2_-)].

#### (Z)-1-(Octadec-9-en-1-yl)-piperidine (cy6T)

Intermediate I (500 mg, 1.32 mmol) prepared in step 1 above was dissolved in 50 mL of ethyl acetate in a 100 mL round-bottom ﬂask. Piperidine (221mg, 2.64mmol) and K_2_CO_3_ (365 mg, 2.62 mmol) were added to the above solution. Reaction was stirred at 80°C for 24 h. The reaction mixture was diluted with 100 mL of ethyl acetate, and washed with water (2 x 100 mL). The organic layer was separated, dried over anhydrous sodium sulfate, and concentrated by rotary evaporator. The residue, upon column chromatographic puriﬁcation over 60-120 mesh size silica gel column using 4% methanol/chloroform (v/v) as eluent, afforded the pure title Lipid 3 as a sunset yellow liquid (378mg, 85.4% yield, Rf=0.3, in 5% methanol:chloroform).

^1^H NMR (300 MHz, CDCl_3_): δ 0.88 (t, 3H, -CH_3_, *J* = 7.0 Hz), 1.26-1.40 [(m, 24H, -(CH_2_)_12_-)], 1.50-1.60 [(m, 6H, -(CH_2_)_2_-], 2.16-2.22 [(m, 4H, -CH_2_-(CH=CH)-CH_2_-)], 2.42-2.56 [(m, 4H, -(CH_2_)_2_-*N*)], 3.48 (t, 2H, N-CH_2_, *J* = 7.8 Hz), 5.36-5.46 [(m, 2H, -CH_2_-(CH=CH)-CH_2_-)].

#### 1,1-Di-((Z)-octadec-9-en-1-yl) piperidin-1-ium iodide (cy6)

Intermediate I (500 mg, 1.32 mmol) prepared in step 1 above was dissolved in 50 mL of ethyl acetate in a 100 mL round-bottom ﬂask. Piperidine (55.25mg, 0.66mmol) and K_2_CO_3_ (91.25 mg, 0.66 mmol) were added to the above solution. Reaction was stirred at 80°C for 96 h. The reaction mixture was diluted with 100 mL of ethyl acetate, and washed with water (2 x 100 mL). The organic layer was separated, dried over anhydrous sodium sulfate, and concentrated by rotary evaporator. The residue, upon column chromatographic puriﬁcation over 60-120 mesh size silica gel column using 8% methanol/chloroform (v/v) as eluent, afforded the pure title Lipid 4 as a yellow solid (378mg, 85.4% yield, Rf=0.1, in 5% methanol:chloroform).

^1^H NMR (300 MHz, CDCl_3_): δ 0.88 (t, 6H, -CH_3_, *J* = 7.2 Hz), 1.24-1.40 [(m, 44H, -(CH_2_)_12_-)], 1.60-1.78 [(m, 10H, -(CH_2_)_2_-], 2.15-2.23 [(m, 8H, -CH_2_-(CH=CH)-CH_2_-)], 3.22-3.62 [(m, 8H, -(CH_2_)_4_-*N*)], 5.30-5.42 [(m, 4H, -CH_2_-(CH=CH)-CH_2_-)].

### 4. Preparation of vehicle formulations

Solutions of Caffeine, ß-estradiol, Spantide-II, α-MSH and Melatonin were prepared respectively with each of the synthesized permeation enhancing lipids. Briefly, accurately weighed amount (1mg) of each drug was dissolved in 10mM vehicle mixture comprising the respective synthesized lipids (cy5, cy5T, cy6 and cy6T) and transcutol.

Based on the preliminary permeation of the various drugs from the solution formulations, the lipid, 1,1-Di-((*Z*)-octadec-9-en-1-yl) piperidin-1-ium iodide (cy5) was selected as the permeation enhancer for the formulation of the lipid-ethanol-drug nanoparticles (LEDs). 

All permeation studies were carried out in comparison to either of the standard permeation enhancers, oleic acid (OA) (20mM) or N-methyl 2-pyrrolidone (NMP) (20mM).

### 5. Preparation of Lipid-Ethanol-Drug Nanoparticles (LEDs)

The nanoparticles (LEDs) were formulated using the modified ethanol injection method. Briefly, 6.51mg of the lipid, 1, 1-Di-((*Z*)-octadec-9-en-1-yl) piperidin-1-ium iodide (cy5) was dissolved in 80μl of absolute ethanol and the solution was rapidly injected into 920μl of magnetically stirred water. The drug to be encapsulated was present in the ethanol solution or in the water, depending on its relative solubility. Nanoparticle vesicle formation was evident upon the appearance of the characteristic opalescence of the colloidal dispersions.

The lipid nanoparticles were used for all subsequent comparative skin permeation studies carried out with standard enhancers.

### 6. Physicochemical characterization of LEDs

The mean particle size and polydispersity index (PI) of LEDs were determined using the Nicomp 380 ZLS particle sizer (Agilent Technologies, Santa Clara, CA, USA), which employs the phenomenon of dynamic light scattering to measure the particle size distribution. The zeta potential (ζ potential, mV) values of the nanoparticles were evaluated using the Nicomp 380 ZLS analyzer via the particle electrophoretic mobility. Each measurement was carried out in triplicate.

### 7. *In vitro* percutaneous permeation studies

#### Preparation of human skin

Dermatomed human skin employed for the in vitro permeation studies was purchased from Platinum Training (USA) and transported in normal saline containing 10% glycerol. The skin was harvested from the thigh, arms and abdomen of a single donor (age 65 years). Biopsies were carried out within 24 hrs post mortem. The skin was then stored at –80°C and used within a week. The dermatomed human skin was thawed and washed in PBS (pH 7.4) for 30 min to remove the excess glycerol prior to the experiments.

#### Preparation of rat skin

The rat skin was collected from CD^®^(SD) hrBi hairless rats, which were sacrificed by an overdose of halothane anesthesia. The skin from the dorsal region was excised with subsequent removal of the subcutaneous fat and connective tissues. Briefly, the collected skin was rinsed with physiological saline, soaked in 10% v/v glycerol in saline solution for 30 min and stored at -80 0C until used. The skin was rinsed in PBS (pH 7.4) for 30 min prior to use. The skin was employed for permeation studies and analyzed by fluorescence microscopy.

#### Skin Permeation set-up

The rat skin was mounted on Franz diffusion cells (Permegear Inc., Riegelsville, PA, USA) between the donor and receiver compartments with the epidermis facing the donor compartment. The receiver compartment comprised of distilled water, stirred at 300 rpm and maintained at 32 °C ± 0.5 °C using a circulating water bath. The mounted skin was equilibrated with the receiver compartment for 1 hr prior to the application of the respective formulations. The skin permeation studies were performed for 24 h under unocclusive conditions. 

#### Application of formulations and permeation studies

For the permeation studies carried out with dermatomed human skin, 100 µl of each of the test formulation was applied on the diffusional surface of the skin in the donor compartment. 300 μl samples were collected from the receiver compartment at different time points from 1-24 h. The receiver compartment samples collected were analyzed using HPLC (Waters Corp, Milford, MA). For the skin collection, after pre-determined time period, the donor cell was removed and the excess formulation was removed from the surface of the skin using a cotton swab. The skin was then washed with 50% v/v ethanolic solution in water and blotted dry with lint-free absorbent wipes. The entire dosing area (0.636 cm^2^) was collected with a biopsy punch. 

The *in vitro* human skin permeation was performed for drug (Caffeine, α-MSH, Melatonin, Spantide II and ß-Estradiol) containing lipid solutions and LEDs. .

For red fluorescence microscopic image analysis, full thickness rat skin was used. Briefly, an accurate amount of the dye was incorporated into LEDs and 130 µl of Rhodamine-LEDs was applied to the skin with permeation studies carried out for 24 hrs. The collected skin was subjected to the same processes indicated above. Solution formulation comprising the dye dissolved in cy5-lipid vehicle solution was maintained as the control.

#### Skin extraction of the drug

To determine the drug amounts retained in the dermatomed human skin samples, extraction of the respective drugs from skin was carried out. The epidermis containing SC (SC + Epi) was separated from the dermis using sharp forceps. The collected skin layers were minced with subsequent addition of 250 µl of PBS (pH 7.4) and boiling in a water bath for 10 min. The samples were cooled down to room temperature and then 250 µl of acetonitrile was added. The vials were sonicated in a bath sonicator for 30 sec and then vortexed for 2 min. Finally, all the tissue samples were centrifuged at 13,000 rpm for 15 min. The supernatant was collected and analyzed for drug content using HPLC.

### 8. HPLC analysis

#### HPLC method for Spantide II

The mobile phases used for Spantide II (SP) were 0.1% v/v TFA in water (solvent A) and 0.1% v/v TFA in acetonitrile (solvent B) and they were run at a gradient of 60:40 to 40:60 (solvent A:B, respectively) for 20 min, with a flow rate of 1.2 ml/min. SP content in the samples was determined at 230 nm. 

#### HPLC method for α-MSH

The mobile phases used for α-MSH were 0.1% v/v TFA in water (solvent A) and 0.1% v/v TFA in acetonitrile (solvent B) and they were run at a gradient of 80:20 to 60:40 (solvent A:B, respectively) for 20 min, with a flow rate of 1.2 ml/min. α-MSH content in the samples was determined at 230 nm.

HPLC system (Waters Corp, Milford, MA) along with a Vydac reverse phase C18 (300 Å pore size silica) analytical column (5 μm, 4.6 × 250 mm) (GraceVydac, Columbia, MD) were used for the analysis of spantide II (SP) and alpha-MSH.

#### HPLC method for caffeine

The mobile phases used for analysis of caffeine were methanol (solvent A) and 0.1% v/v TFA in water (solvent B) and they were run at a gradient of 20:80 to 85:15 (solvent A:B, respectively) for 10 min, with a flow rate of 1 ml/min. Caffeine content in the samples was determined at 272 nm.

#### HPLC method for Melatonin

The mobile phase used for analysis of melatonin was methanol:water (60:40) with a flow rate of 0.5 ml/min. Melatonin content in the samples was determined at 223 nm.

#### HPLC method for ß-estradiol

The mobile phases used for analysis of estradiol were acetonitrile (solvent A) and water (solvent B) and they were run at a gradient of 30:70 for 20 min, then 80:20 for 23 min followed by 30:70 (solvent A:B, respectively) for 25 min, with a flow rate of 1.2 ml/min. Estradiol content in the samples was determined at 240nm.

HPLC system (Waters Corp, Milford, MA) and waters symmetrical C18 analytical column (5 μm, 4.6 × 250 mm) were used for the analysis of caffeine, melatonin, and ß-estradiol. 

### 9. Skin imaging studies

Microscopic image analysis was carried out using the Olympus BX40 microscope equipped with computer-controlled digital camera (DP71, Olympus Center Valley, PA, USA) (10X objective) to establish the distribution of Rhodamine dye across the skin layers. The protocol followed for microscopic imaging analysis was that explained by Shah et al. Full thickness and lateral skin sections were obtained via cryosectioning up to a depth of 380 μm with a cryotome (Shandon, England). The skin sections were visualized and analyzed for red fluorescence for all the samples.

### 10. Statistical analysis

The results have been expressed as the mean ± S.D for at least three replicates. The comparison between the multiple groups has been established by a one-way analysis of variance (ANOVA) and between two groups by student’s t test analysis. A p value less than 0.05 (p <0.05) was considered statistically significant.

## Results

### 1. Chemistry

Toward developing the chemical permeation enhancers for the transdermal drug delivery, we designed lipids 1- 4 such that lipids 1-2 and 3-4 architecturally differed only in head group regions. Lipid 1 was synthesized by coupling oleyl iodide with pyrrolidine. Lipid 2 was synthesized by quarternizing pyrrolidone with 2 equivalents of oleyl iodide. Lipid 3 was synthesized by coupling oleyl iodide with piperidine. Lipid 4 was synthesized by quarternizing piperidine with 2 equivalents of oleyl iodide. The structures of all lipids 1-4 were confirmed by ^1^H NMR spectral analysis.

### 2. Preparation of vehicle formulations

To enhance the percutaneous permeation of the drugs, solutions of Caffeine, ß-estradiol, Spantide-II, α-MSH and Melatonin were prepared respectively with each of the synthesized permeation enhancing lipids. Briefly, accurately weighed amount (1mg) of each drug was dissolved in 10mM, 1ml vehicle mixture comprising the respective synthesized lipids (cy5, cy5T, cy6 and cy6T) and transcutol. Each formulation was clear and transparent without the formation of precipitates observed at any instance.

### 3. *In vitro* permeation studies with vehicle formulations and HPLC analyses

**Figure 1:**
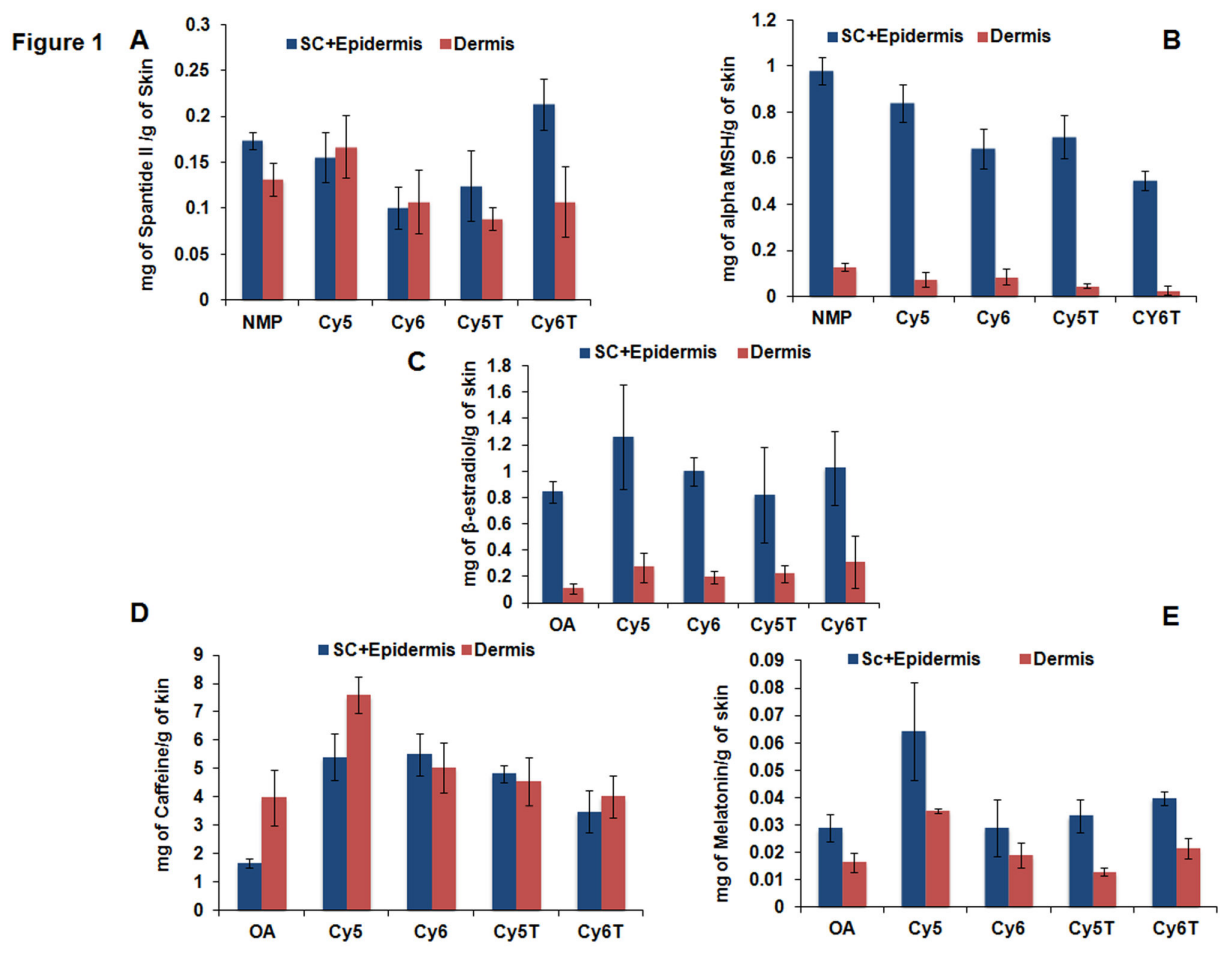
*In* *vitro* skin permeation studies with cy5, cy6, cy5T and cy6T vehicle formulations in dermatomed human skin with NMP and OA employed as reference permeation enhancers. Data represent mean ± SD, n=6. Data shows comparable skin retention in comparison to NMP for the peptide drugs, A) Spantide II and B) α-MSH but improved skin retention of the small molecular drugs, C) β-estradiol, D) caffeine and E) melatonin in comparison to OA.

**Figure 2:**
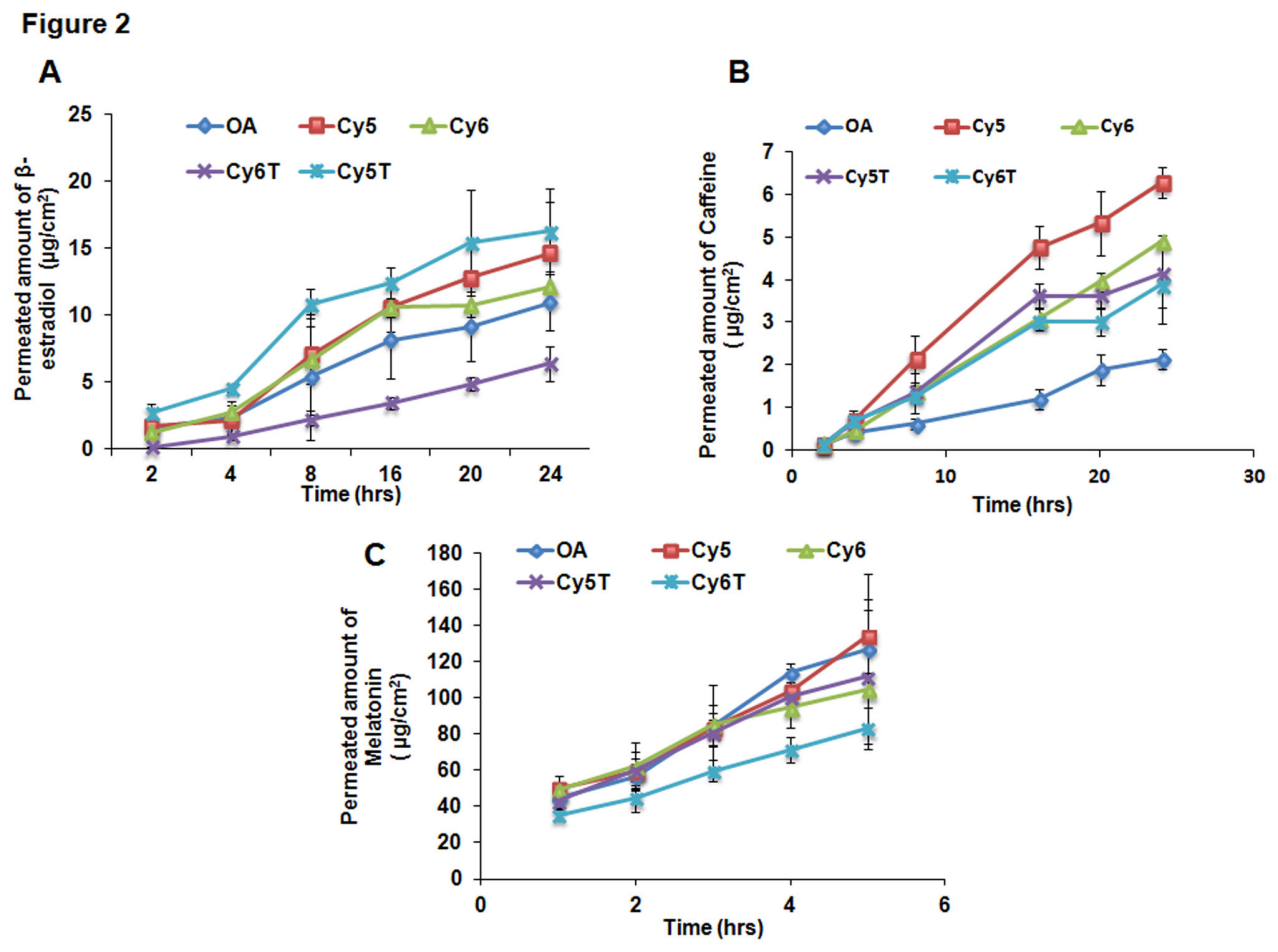
*In* *vitro* skin permeation studies with cy5, cy6, cy5T and cy6T vehicle formulations with dermatomed human skin demonstrating amount of drug (μg/cm^2^) permeating into the receiver compartment over 24 hrs. Detection was possible for only the small molecular drugs, A) β-estradiol, B) caffeine and C) melatonin in comparison to the peptide drugs. Data represent mean ± SD, n=6.

For the screening of the lipids for the most efficient permeation enhancer, different vehicle formulations containing the respective lipids were employed for skin permeation studies for 24 hrs. The studies revealed that after 24 hr permeation studies, all the lipids exhibited proficient transport of drug cargo across the stratum corneum into the epidermal and dermal layers comparable to the standard permeation enhancers, NMP and OA (Figure 1). There was increase in drug permeation into the receiver compartment with increase in time for some of the drugs (Figure 2). Additionally, cy5 lipid exhibited relatively, the highest drug retention in the epidermal and dermal layers of the skin for each of the drugs examined. However, the increase in drug permeation and skin retention was not significant (p>0.05) for any of the vehicle formulations.

#### Permeation of Spantide II

For permeation with the peptide drug, Spantide II (Figure 1A), there was no amount of drug detected in the receiver compartment for both the lipid vehicle formulations and the standard enhancer, NMP. However, drug retention at the SC+Epidermis was 0.16 ± 0.03, 0.10 ± 0.02, 0.12 ± 0.04 and 0.21 ± 0.03 mg per g of skin for cy5, cy6, cy5T and cy6T lipids, respectively compared to NMP with drug retention of 0.17 ± 0.01 mg per g of skin. At the dermal layer, drug retention was 0.17 ± 0.03, 0.11 ± 0.03, 0.09 ± 0.01 and 0.11 ± 0.04 mg per g of skin for cy5, cy6, cy5T and cy6T lipids, respectively whilst NMP showed drug retention of 0.13 ± 0.02 mg per g of skin. Hence, cy5 lipid showed superior permeation compared to the other lipids with about 1.3-folds more drug retention in the dermal layer but 1.1-folds less in the SC+Epidermis, compared to NMP. This suggested that the cy5 lipid was capable of overcoming the lipid bilayer permeation barrier of the stratum corneum to transport the Spantide II into the deeper layers of the skin.

#### Permeation of α-MSH

Permeation results for the second peptide drug, α-MSH revealed similar results as noted for Spantide II (Figure 1B). There was no detection of the drug in the receiver compartment for any of the lipids and NMP. On the other hand, there were variable amounts of α-MSH retained at the different layers of the skin. For the SC+Epidermal layer, drug retention was 0.84 ± 0.07, 0.64 ± 0.09, 0.69 ± 0.05 and 0.51 ± 0.03 mg per g of skin for cy5, cy6, cy5T and cy6T lipids, respectively and 0.98 ± 0.06 mg per g of skin for NMP. For the dermis drug retention, the amounts of drug detected were 0.07 ± 0.03, 0.09 ± 0.03, 0.05 ± 0.01 and 0.03 ± 0.02 mg per g of skin for cy5, cy6, cy5T and cy6T lipids respectively and 0.13 ± 0.02 mg per g of skin for NMP. It was hence evident that cy5 demonstrated greater permeation of the peptide drug into the epidermal layer in comparison to the other lipids studied. The permeation of α-MSH by cy5 lipid was however less than NMP at both the epidermal and dermal layers.

#### Permeation of β-estradiol

For the permeation studies carried out with β-estradiol, some amount of drug was detected in the receiver compartment for all the lipids and OA, the standard permeation enhancer used (Figure 2A). There was gradual increase in amount of drug detected with the increase in time of incubation for all the lipids examined. There was also the illustration of sustained release of drug into the receiver compartment for each lipid vehicle formulation. The vehicle formulation of cy5T exhibited the greatest amount of drug permeation into the receiver compartment at all the time points whilst the vehicle formulation of cy6T showed the least drug permeation at all the time points. Drug permeation for cy5 lipid observed was more enhanced than all the other lipids with the exception of the cy5T lipid. In addition, the vehicle formulations comprising the respective lipids, cy5, cy5T and cy6, demonstrated superior drug permeation into the receiver compartment compared to the standard enhancer, OA, except the cy6T lipid. At 24 hrs, drug permeation into the receiver compartment was 14.64 ± 3.80, 12.14 ± 1.09, 16.26 ± 3.18 and 6.38 ± 1.30 μg/cm^2^ and 10.94 ± 2.13 μg/cm^2^ for cy5, cy5T, cy6, cy6T and OA, respectively.

For drug retention in the SC+Epidermal layer (Figure 1C), cy5 lipid demonstrated the highest amount of 1.26 ± 0.40 mg/g of skin compared to all the other lipids. This was about 1.5-fold more than the amount for OA, which showed drug retention of 0.84 ± 008 mg/g of skin. The amount of drug retained for the other lipids were 082 ± 0.36, 1.00 ± 0.11 and 1.02 ± 0.28 mg/g of skin for cy5T, cy6 and cy6T, respectively. For the dermal layer, drug retention for cy5 lipid was more enhanced than the other lipids and standard permeation enhancer, OA, but less compared to cy6T. At the dermal layer, cy5 enhanced pronounced drug retention (2.5-fold) more than OA and 1.2 and 1.4-folds more than cy5T and cy6, respectively. In comparison to cy6T, drug retention was relatively 1.1-fold decreased for cy5 lipid. Overall, the drug amounts retained in the dermal layer were determined as 0.27 ± 0.11, 0.22 ± 0.07, 0.19 ± 0.05 and 0.31 ± 0.20 mg/g of skin for cy5, cy5T, cy6 and cy6T respectively and 0.11 ± 0.04 mg/g of skin for OA.

#### Permeation of Caffeine

Analysis carried out demonstrated that there was permeation of drug into the receiver compartment for all the lipids as well as for the standard permeation enhancer, OA (Figure 2B). There was an increase in the amount of drug permeating with time up to the 24 hr incubation period carried out for the permeation studies in each formulation treatment group. The drug release profile observed for each lipid vehicle formulation was of sustained release characteristics though with sharp onset of release. However, cy5 lipid demonstrated a more rapid release of drug up to 16 hrs, after which the release was more gradual and sustained. In addition, at 24 hrs, cy5 lipid demonstrated the highest amount of caffeine drug permeated into the receiver compartment compared to the others. This amount, determined to be 6.29 ± 0.35 μg/cm^2^ for cy5 was about 2.9-fold more than the amount detected for OA (2.14 ± 0.24 μg/cm^2^). In addition, the other lipids, cy6, cy5T and cy6T showed 2.29, 1.93 and 1.81-fold increase in drug permeation respectively, in comparison to OA at 24 hrs. However, cy5 vehicle formulation showed 1.28, 1.52 and 1.62-folds increase in amounts detected in the receiver compartment more than cy6, cy5T and cy6T lipids, respectively, which revealed permeation amounts of 4.90 ± 0.14, 4.14 ± 0.79 and 3.88 ± 0.91 μg/cm^2^, respectively.

Further, analysis of the skin revealed that cy5 lipid caused the highest skin retention of caffeine in both the epidermal and dermal layers, compared to the other lipids (Figure 1D) whilst the permeation enhancer standard, OA, showed the lowest amount of caffeine retained. In comparison, cy5 lipid vehicle formulation caused about 3.26 and 1.91-fold increase in amount of drug retained at the epidermal and dermal layers, respectively more than OA. The skin retention of caffeine at the epidermal layer for cy6 lipid was comparable to cy5 lipid. However, amount of drug retained in the dermis was about 1.5-fold less than cy5 lipid. The respective amounts of caffeine determined in the epidermis were 5.41 ± 0.82, 1.66 ± 0.17, 5.50 ± 0.74, 4.83 ± 0.30 and 3.48 ± 0.74 mg per g of skin for cy5, OA, cy6, cy5T and cy6T respectively. For the dermal skin retention, the amounts of caffeine determined were 7.58 ± 0.64, 3.97 ± 0.99, 5.04 ± 0.89, 4.56 ± 0.84 and 4.02 ± 0.74 mg per g of skin for cy5, OA, cy6, cy5T and cy6T, respectively.

#### Permeation of Melatonin

The analysis carried out to determine the amount of melatonin in the receiver compartment has been illustrated in Figure 2C. It was observed that there was melatonin drug detected in the receiver compartment for all the lipid vehicle formulations at each time point of the analysis up to 5 hours, beyond which no detection was observed. In addition, there was increase in the amount of drug detected with increase in duration of time. Overall, melatonin showed the highest permeation into the receiver compartment for the time points at which the drug was detected, compared to all the other drugs. At 5 hrs, cy5 lipid demonstrated the maximum permeation of melatonin of 134 ± 20.17 μg/cm^2^, which was about 1.06-fold more in comparison to OA with permeation of 126.67 ± 41.48 μg/cm^2^. On the other hand, cy6T revealed the least permeation of 83.09 ± 11.35 μg/cm^2^ (1.61-fold less in comparison to cy5 lipid). The permeation amounts for the other lipids were 104.32± 20.42 and 111.88 ± 36.88 μg/cm^2^ for cy6 and cy5T, respectively.

Skin retention of drug observed for all lipids reinforced earlier observations that cy5 is superior to all the other lipids synthesized as well as to the permeation enhancer, OA, in effecting the permeation of all the drugs examined (Figure 1E). Cy5 demonstrated the highest skin retention of melatonin in the epidermis and dermis with amounts of 0.06 ± 0.02 and 0.04 ± 0.0008 mg per g of skin, respectively. These amounts were about 2-fold more than that for OA, which revealed skin retention amounts of 0.03 ± 0.005 and 0.02 ± 0.004 mg of melatonin per g of skin for the epidermal and dermal layers, respectively. The skin retention values for cy6, cy5T and cy6T vehicle formulations were 0.03 ± 0.01, 0.03 ± 0.006 and 0.04 ± 0.003 mg per g of skin respectively for the epidermal layer whilst for the dermal layer, the amounts of drug were 0.02 ± 0.005, 0.01 ± 0.002 and 0.02 ± 0.004 mg per g of skin, respectively.

### 4. Physicochemical characterization of LEDs

Due to the superior and enhanced skin permeation properties of cy5 lipid in comparison to the other lipids, it was selected for the preparation of the lipid-ethanol-drug nanoparticles (LEDs). The nanoparticles exhibited stable nanodispersion and uniform size for all the drugs employed. Caffeine-LEDs exhibited the smallest average particle size of 140.2 nm with a polydispersity of 0.21 whilst Melatonin-LEDs demonstrated the largest particle size range with average size of 198.2 nm and polydispersity of 0.34. All the drug nanoparticles demonstrated largely positive zeta potentials because of the cationic characteristic of the cy5 lipid, which impacted its positive charges on the nanoparticles formed. The drug entrapment efficiencies of the nanoparticles were determined to be in the range of 60 to 75%. β-estradiol-LEDs were determined to have the highest entrapment efficiency of 74.3 % whilst Caffeine-LEDs had the least with entrapment efficiency of 60.8%. The average particle sizes, zeta potentials and drug entrapment efficiencies have been represented in Table 1.

**Table 1:** Representation of the characterization of the cy5 lipid ethanolic nanoparticles including the particle size, zeta potential and entrapment efficiency.

**Formulation**	**Particle Size (nm)**	**PDI**	**Zeta Potential (mV)**	**Entrapment Efficiency (%)**
**αMSH-LEDs**	**158.3**	**0.35**	**23.5 ± 4**	**62.3**
**Spantide-LEDs**	**187.2**	**0.42**	**21.3 ± 2**	**71.2**
**β-estradiol-LEDs**	**167.4**	**0.51**	**22.7 ± 6**	**74.3**
**Melatonin-LEDs**	**198.2**	**0.34**	**26.2 ± 3**	**68.4**
**Caffeine-LEDs**	**140.2**	**0.21**	**31.2 ± 4**	**60.8**

Data represent mean ± SD, n=3.

### 5. *In vitro* permeation studies with LEDS, skin extraction and HPLC analyses

**Figure 3:**
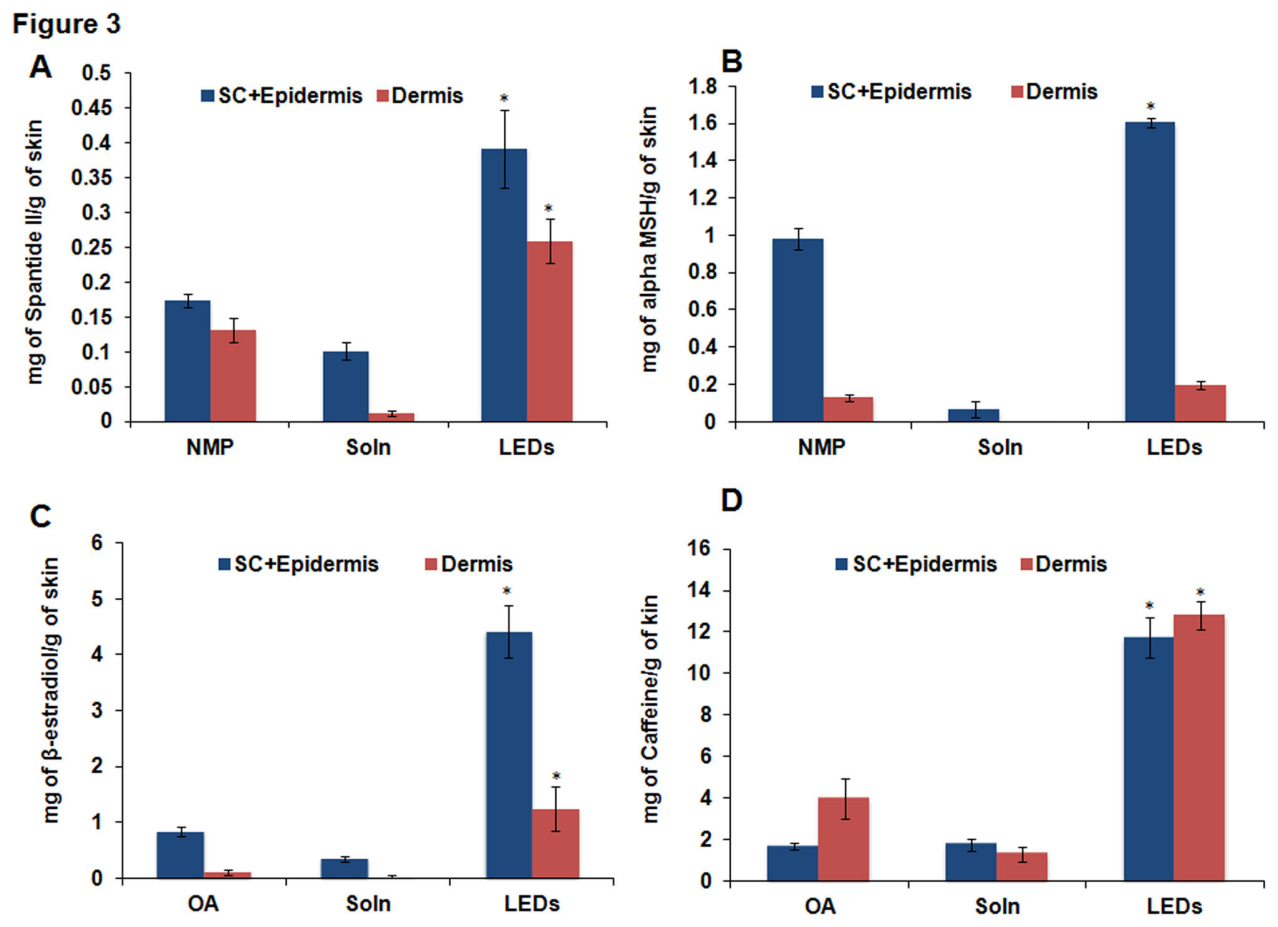
*In* *vitro* permeation studies with cy5 lipid ethanolic nanoparticles in dermatomed human skin in comparison to the reference permeation enhancers, NMP and OA. The results depict the amount of the drug retained in the stratum corneum & epidermal and dermal layers, respectively for A) Spantide II, B) α-MSH, C) β-estradiol and D) caffeine. Data represent mean ± SD, n=6. p value less than 0.05 (p <0.05) was considered statistically significant.

**Figure 4:**
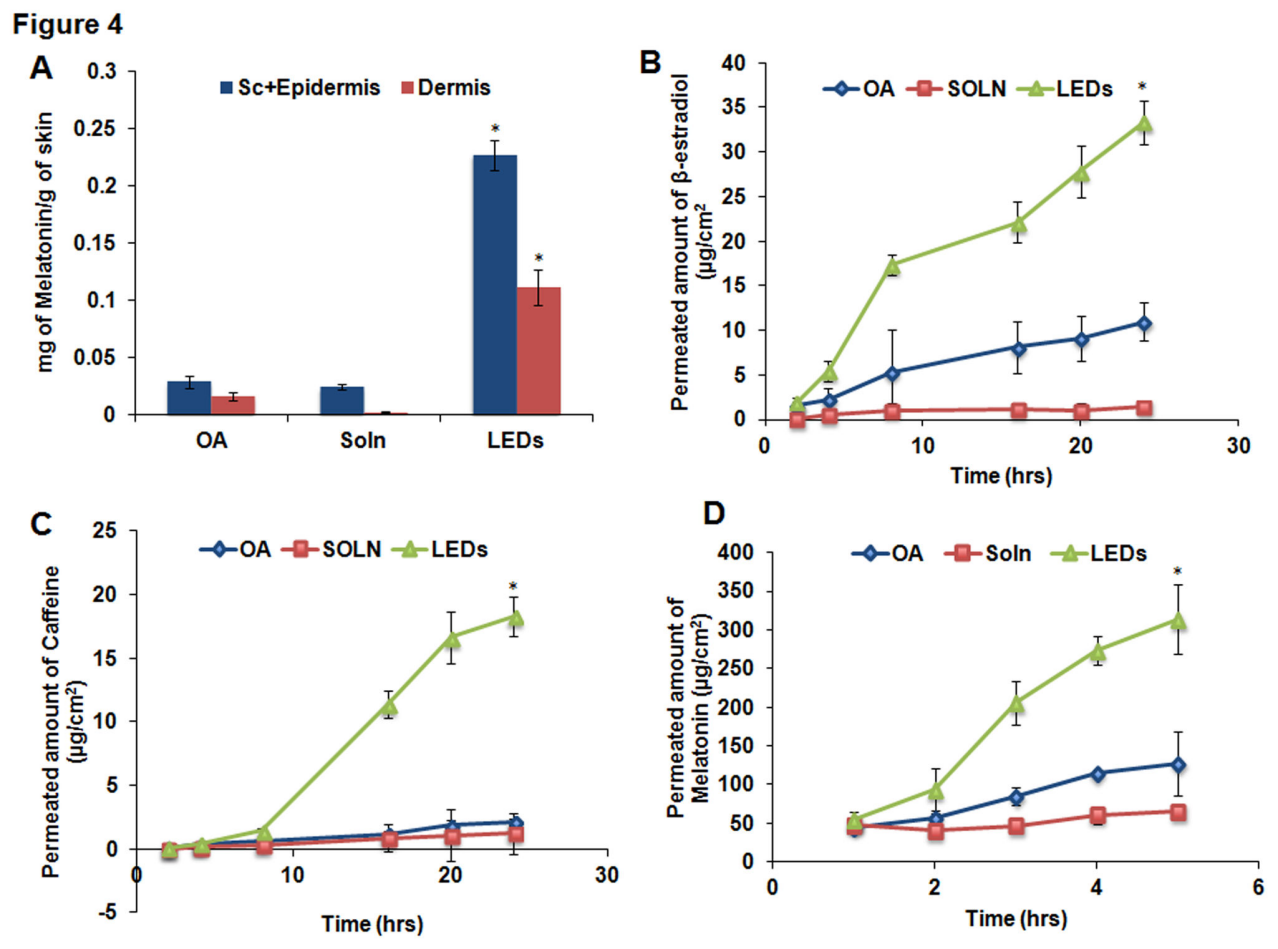
*In* *vitro* skin permeation studies with dermatomed human skin demonstrating A) skin retention of melatonin in the stratum corneum & epidermal and dermal layers, respectively and B) amount of drug (μg/cm^2^) permeating into the receiver compartment over 24 hrs with cy5 lipid ethanolic nanoparticle formulation. As noted for the vehicle formulations, detection was possible for only the small molecular drugs, A) β-estradiol, B) caffeine and C) melatonin in comparison to the peptide drugs. Data represent mean ± SD, n=6. p value less than 0.05 (p <0.05) was considered statistically significant.

The in vitro permeation studies carried out with the Lipid-ethanolic-drug nanoparticles (LEDs) showed enhanced permeation and skin retention of drug in the deeper layers compared to the standard permeation enhancers and drug vehicle solution for each of the drugs studied (Figure 3 & 4). The nanoparticles had the capacity to overcome the skin barriers to increase the permeation of caffeine, β-estradiol and Melatonin into the receiver compartment more effectively than either of OA or SOLN. However, no drug was detected in the receiver compartment for the Spantide and α-MSH. Caffeine-LEDs exhibited the greatest skin retention of drug at both the epidermal and dermal layers, compared to all the other drugs. 

For Spantide-LEDs (Figure 3A), there was significant (p<0.05) increase in skin retention of spantide drug at both the epidermal layer and dermal layers compared to both NMP and SOLN. At the epidermal layer, there were about 2.26 and 3.87-folds more respectively. At the dermal layer, there were 1.97 and 23.32-folds increase in drug amount detected for the nanoparticles compared to NMP and SOLN, respectively. At the end of the 24 hr permeation studies, the determined amounts of drugs were 0.39 ± 0.06, 0.17 ± 0.01 and 0.10 ± 0.01 mg of caffeine per g of skin for LEDs, NMP and SOLN respectively at the epidermal layer. Drug amounts at the dermal layer were 0.26 ± 0.03, 0.13 ± 0.02 and 0.01 ± 0.004 mg per g of skin for LEDs, NMP and SOLN, respectively.

α-MSH-LEDs revealed similar results noted above for Spantide (Figure 3B). There was significant (p<0.05) drug retention at the epidermal layer compared to both NMP and SOLN. There was 1.6 ± 0.03, 0.98 ± 0.06 and 0.06 ± 0.04 mg of drug retained per g of skin at the epidermal layer for LEDs, NMP and SOLN, respectively whilst for the dermal layer, drug amount retained was 0.20 ± 0.02, 0.13 ± 0.02 and 0.003 ± 0.0004 mg per g of skin for LEDs, NMP and SOLN respectively.

The nanoparticles for β-estradiol permeated deeper into the skin to deliver the drug, which was detected in substantial amount at the dermal layer (Figure 3C). Significant (p<0.05) amount of drug was detected in the receiver compartment for β-estradiol-LEDS but very minimal amounts for SOLN (Figure 4B). Release of drug for OA was more gradual without significant increase in drug concentration over the entire 24 hrs. However, for LEDs, there was rapid but sustained release of drug from the start of the permeation studies up to 24 hrs. At 24 hrs, the amount of drug detected for LEDs was 33.34 ± 2.42 μg/cm^2^, about 3.05-folds more than OA with permeation amount of 10.94± 2.13 μg/cm^2^ and about 23.65-folds more than SOLN with permeation amount of 1,41 ± 0.62 μg/cm^2^. Furthermore, amount of β-estradiol delivered into and retained in the dermal layer by LEDs (1.25 ± 0.40 mg per g of skin) was significantly (p<0.05) more than OA (0.11 ± 0.04 mg per g of skin) by 11.36-folds and SOLN (0.03 ± 0.02 mg per g of skin) by 41.67-folds more. The epidermal layer drug amounts retained were 4.41 ± 0.48, 0.84 ± 0.08 and 0.34 ± 0.05 mg of β-estradiol per g of skin for LEDs, OA and SOLN, respectively.

For Caffeine-LEDs, there was a relative delayed release of drug until 8 hrs after which there was rapid increase in amount of drug permeating into the receiver compartment up to 24 hrs (Figure 4C). For both OA and NMP, there were very minimal amounts of drug detected throughout the 24 hr permeation studies. The amount of drug detected in the receiver compartment at 24 hrs for the nanoparticles was significantly (p<0.05) more, about 8.57 and 14.81-folds more than OA and SOLN, respectively. The amounts of drug permeated were 18.31 ± 1.59, 2.14 ± 0.24 and 1.24 ± 0.29 μg/cm^2^ for LEDs, OA and SOLN, respectively. Additionally, there were about 7.07 and 6.72-folds more of the caffeine retained at the epidermal layer for the nanoparticles in comparison to OA and SOLN, respectively whilst the nanoparticles enhanced drug permeation into the dermal layer by 3.22 and 9.80-folds more than OA and SOLN, respectively. In both instances, skin retention was determined to be significantly (p<0.05) greater in comparison to NMP and SOLN, respectively. The amounts of drug retained in the skin were determined to be 11 ± 0.96, 1.66 ± 0.17 and 1.75 ± 0.32 mg per g of skin for LEDs, OA and SOLN, respectively, in the epidermal layer. At the dermal layer, amounts included 12.80 ± 0.66, 3.97 ± 0.99 and 1.31 ± 0.37 mg per g of skin for LEDs, OA and SOLN, respectively (Figure 3D).

Lastly, the results observed for melatonin were similar to those noted above for the other drugs. There was however an overall increase in drug permeation into the receiver compartment for melatonin up to 5 hrs, as noted previously for the vehicle formulations, delivered by LEDs compared to the other drugs (Figure 4D). There was however significant (p<0.05) difference in the amount of melatonin detected for LEDs compared to OA and SOLN (about 2.47 and 4.84-folds more, respectively). The amounts permeating into the receiver compartment at 5 hrs were determined as 313.93 ± 41.48, 126.67 ± 44.99 and 64.85 ± 4.50 μg/cm^2^ for LEDs, OA and solution, respectively. However, in contrast, the amounts of melatonin detected in the skin were the least compared to the other drugs. This can be attributed to the fact that much of the drug permeated the skin into the receiver compartment delivered by the nanoparticles. The amounts of melatonin retained in the epidermis were 0.23 ± 0.01, 0.03 ± 0.005 and 0.02 ± 0.002 mg per g of skin for LEDs, OA and SOLN, respectively whilst the amounts retained in the dermal layer were 0.11 ± 0.02, 0.005 ± 0.004 and 0.002 ± 0.0003 mg per g of skin for LEDs, OA and SOLN, respectively (Figure 4A). 

### 6. Skin imaging

**Figure 5:**
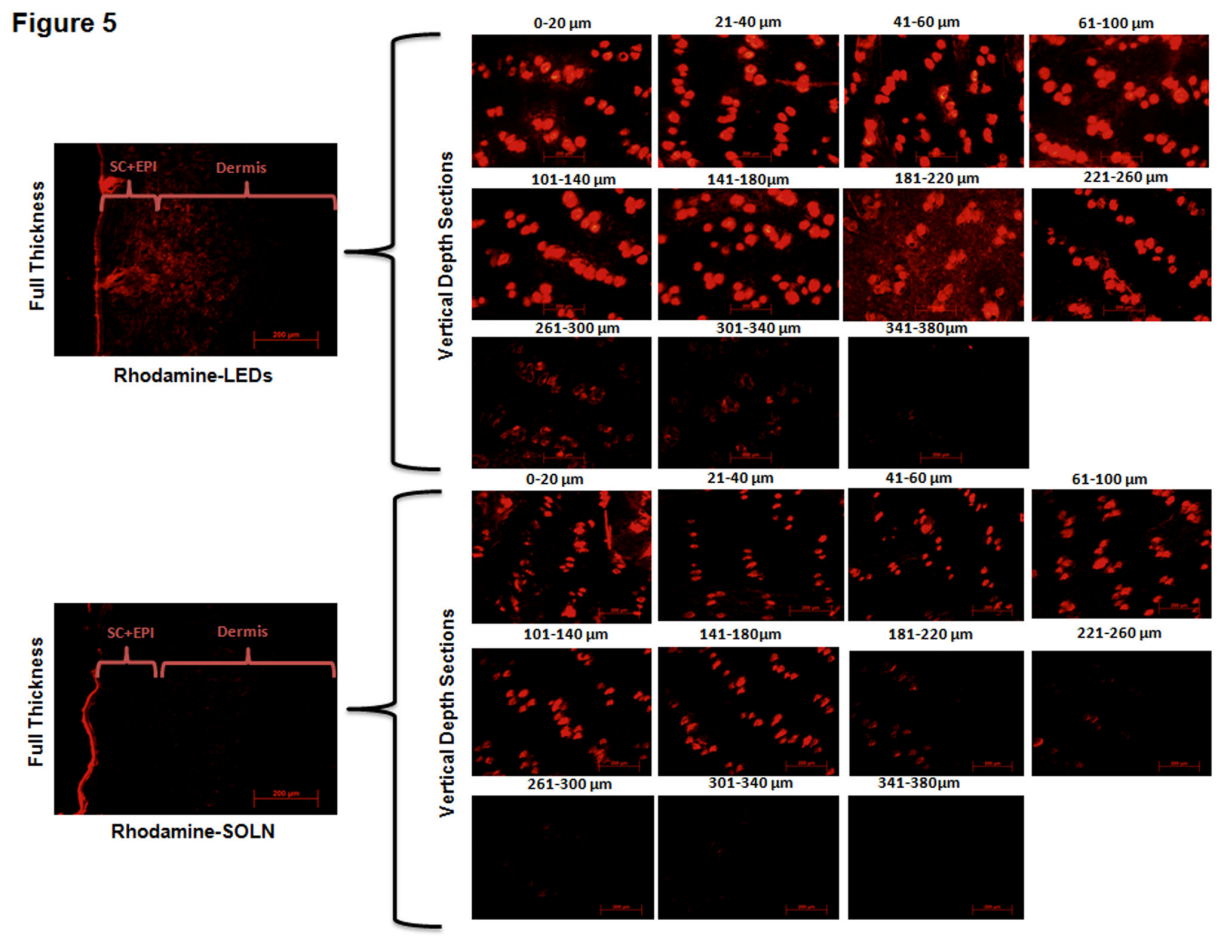
Fluorescence microscopic data analysis in *in* *vitro* skin permeation studies with full thickness rat skin. Data depicts permeation of Rhodamine across the skin barrier into the dermal layer delivered by LEDs in comparison to cy5-vehicle solution. Data represent mean ± SD, n=6. p value less than 0.05 (p <0.05) was considered statistically significant.

The fluorescence microscopic analysis indicated that the LEDs had the capability to permeate into the deeper layers of the skin whilst carrying the rhodamine-PE as cargo across the skin barrier (Figure 5). This was revealed in the detection of red fluorescence up to skin depth of 340 μm for Rhodamine-LEDs compared to solution, which showed fluorescence only up to skin depth of 200 μm. For the nanoparticles, there was significant (p<0.05) increase in fluorescence detected at skin depth sections from 0 μm up to 220 μm, where maximum fluorescence was observed. However, there was diminished fluorescence detected from 221 μm to 340 μm, where very minimal fluorescence was observed. On the other hand, for solution, fluorescence was detected from skin depth of 0 μm to 180 μm, after which very minimal detections of fluorescence were observed for depth sections from 181 μm to 260 μm with trace amounts at 261-300 μm. There was however, no detection of fluorescence for skin depths of 301 μm to 380 μm for solution.

## Discussion

Currently, hypodermic needles are the only available modes for systemic delivery of macromolecular drugs into humans. Transdermal delivery offers an attractive alternative to needle based drug administration because it is noninvasive. There is however limitation to the extent of transdermal permeation of relatively large drugs such as peptides (molecular mass > 500 Da), which represent a large majority of active agents for therapeutic applications, across the stratum corneum [18]. Several chemical penetration enhancers (CPEs) have been identified to perturb the SC barrier to facilitate molecular delivery. A large amount of transdermal literature is available for studying and predicting the effect of chemical enhancers on skin permeation [19,20]. However, incorporation of CPEs into products has been mitigated by safety concerns related to the distortion of the integrity of the skin membrane [21,22]. Accordingly, overcoming the skin barrier in a safe and effective way still remains the bottleneck of transdermal and topical therapies.

Pyrrolidones have been used as permeation enhancers for numerous molecules including hydrophilic (e.g. mannitol and 5-flurouracil) and lipophilic (progesterone and hydrocortisone) permeants. In addition, they have proven to be proficient carriers of peptide molecules such as Spantide II (33) and Cyclosporine A (34) across the stratum corneum into deeper layers of the skin for the treatment of inflammatory conditions such as psoriasis and acute contact dermatitis (ACD). On the other hand, N-methyl-2-pyrrolidone (NMP) has been employed with limited success as a penetration enhancer for captopril formulated into a matrix-type transdermal patch [23]. The pyrrolidones partition well into human stratum corneum within the skin tissue and they may act by altering the solvent nature of the membrane of the lipid bilayer. Further, they have been used to generate reservoirs within the skin membrane. Such a reservoir effect offers a potential for sustained release of a permeant from the stratum corneum over extended time periods [24]. This influenced our decision and choice to employ NMP as a standard permeation enhancer for comparison with our synthesized pyrrolidone lipids for the peptide drugs, Spantide II and α MSH, which were selected for permeation studies (Figures 1 and 2).

In addition, percutaneous drug absorption has been increased by a wide variety of long-chain fatty acids, the most popular of which is oleic acid. It is of interest to note that many penetration enhancers such as azone contain both saturated or unsaturated hydrocarbon chains and some structure - activity relationships have been drawn from the extensive studies of Aungst who employed a range of fatty acids, acids, alcohols, sulphoxides, surfactants and amides as enhancers for naloxone [25]. For example, oleic acid (OA) greatly increased the flux of drugs such as salicylic acid by 28-fold and 5-flurouracil by 56-fold respectively through human skin membrane *in vitro* [26]. OA has been shown to disrupt the orderly arrangement of the lipid domain which comprise mostly of saturated straight chain skin lipids and increases the lipid fluidization in the stratum corneum, hence allowing both small and macromolecules to permeate through [27,28]. Also, OA has been shown to induce remarkably improved skin permeation for melatonin and estradiol with hairless rat skin in vitro [29,30]. OA was hence used as control to evaluate the transdermal permeation efficacies of the novel lipids of small molecule drugs such as ß-estradiol, caffeine and melatonin (Figures 1 and 2). The drugs chosen could also be grouped into the large groups, hydrophilic drugs comprising caffeine, α-MSH and melatonin and hydrophobic drugs comprising of ß-estradiol and spantide II.

Our studies revealed that cy5 lipid was the most efficient at enhancing the permeation and skin retention of the small molecule drugs. In comparison to OA, cy5 also demonstrated superior permeation across the stratum corneum into the dermal skin layer (Figures 1 and 2). However, in comparison to NMP, cy5 lipid exhibited comparable skin retention of the peptide macromolecules at both the epidermal and dermal layers, even though the concentration of NMP utilized for the vehicle formulation was about twice that of cy5 (Figure 1). Higher activity of Cy5 can be explained by the structural similarities of its head group to NMP, and its tail region, which has oleyl chains forming the hydrophobic core. The two hydrophobic oleyl chains might have imparted higher fusogenic behavior to the Cy5 molecule, hence demonstrating increased permeation. Interestingly, nanoparticles prepared with Cy5 lipid (LEDs) were found to be more effective than lipid vehicle formulations in the permeation and skin retention of both small and macromolecular drugs in comparison to oleic acid and NMP, respectively (Figure 3). This observation hence indicated that the water present in LEDs induced transdermal enhanced penetration possibly by expanding the polar head groups of the SC lipid bilayer or by squeezing and distorting the lipid bilayer and causing swelling/engorgement of the corneocytes [31] Thus, it facilitated the LEDs to fuse with and subsequently pass through the lipid bilayers of stratum corneum consequently increasing the permeation. Menon et al. [32] proposed an aqueous pore pathway for the diffusion of active therapeutic agents across the skin under high stress conditions such as excessive hydration. According to this theory, under conditions such as excessive hydration, preexisting scattered lacunae embedded in the lipid bilayer expand and form continuous water channels that facilitate the diffusion of both hydrophilic and lipophilic permeants. This is suggestive of the observed increased permeation of the drug in the presence of cy5-LED than in the vehicle formulation containing the same lipid as permeation enhancer in transcutol.

Superior permeation capacity of LEDs was further investigated in full thickness hairless rat skin treated with ﬂuorescently labeled LEDs (Rhodamine-PE) for 24 hrs. Subsequently, analysis was performed using epifluorescent microscope on the cryosectioned skin depth sections. Figure 5 gives a representation of the skin depth sections after treatment with Rhodamine-Cy5 lipid vehicle solution. The studies revealed that the dye complexes formed were mainly confined to the upper layers of the epidermis of the skin. Also, rhodamine LEDs treated skin showed a bright ﬂuorescent signal in the SC in contrast to Rhodamine Cy5-solution. LEDs showed intense signal in the upper strata and an even more pronounced migration towards deeper layers, as the red ‘clumps’ were located at a distant reach from the SC. Shah et al. [33] have also reported that when Ketoprofen and Spantide II were encapsulated in the PLGA-Chitosan hybrid nanoparticles, skin retention of the drugs was increased significantly compared to when applied in a solution form due to strong affinity of positively charged amine groups (of chitosan) to the skin surface [33]. 

The current study demonstrated that all the novel lipids showed improved permeation of a variety of drugs and particularly, Cy5 showed superior effects. Nanoparticles of Cy5 showed significant increase in drug permeation and skin retention. The enhanced penetration of nanoparticles was as a result of the active interaction between the SC components (corneocytes and lipids) and the novel Cy5 of LEDs. On application of LEDs on the skin surface, the following sequence of events are being proposed to have taken place: (i) ionic interaction between positively charged outer lipid layer of LEDs with negatively charged residues of the proteins and lipids of SC; (ii) possible occurrence of transitional destabilization of the membrane beyond a threshold concentration of LEDs; (iii) formation of a film over the skin surface by LEDs leading to higher hydrating effects that caused SC swelling and opening which allowed higher penetration of permeant and (iv) penetration of encapsulated substances by LEDs possibly through hair follicles and furrows which served as drug reservoirs.

In conclusion, toward developing chemical permeation enhancers, we have designed and synthesized novel lipids containing amine based heterocyclic head group and oleyl fatty acyl chains by making changes in the two parameters in both head and tail regions. We selected pyrrolidinium and pyridinium groups for the constitution of the head groups as they have structural resemblance with the well-known chemical enhancers, 6-aminohexanoates and azones. For the other parameter, the hydrophobic tails were changed to both single and dual chains. Our studies demonstrated that Cy5 lipid with its positive charge was more effective in permeating the drugs across the skin. Further Cy5 based nanoparticle system significantly increased the permeation of the drugs. The permeation of the lipid solution and LEDs were examined using rhodamine-PE permeation across the skin under epifluorescent microscope. 

In summary the present findings demonstrated that i) cationic lipid with 5 membered amine heterocyclic ring showed higher permeating efficacy than the 6 membered amine hertocyclic ring and ii) the nanoparticle system prepared with Cy5 showed significant (p<0.05) increase in the permeation of the drugs than the control penetration enhancers, oleic acid and NMP.
