# Correction: Design, Synthesis of Novel Lipids as Chemical Permeation Enhancers and Development of Nanoparticle System for Transdermal Drug Delivery

**DOI:** 10.1371/journal.pone.0096964

**Published:** 2014-04-29

**Authors:** The PLOS ONE Staff

The authors of this article wish to acknowledge and apologize for the use of text verbatim from other articles used as references for the study without appropriate citation and quotations.

The overlap in the text with that from previous publications relates to the following fragments in the text of the Introduction, Methods and Discussion sections of the article:

1. The Introduction section. There is overlap with text from the articles, “Synthesis, physico-chemical properties and penetration activity of alkyl-6-(2,5-dioxopyrrolidin-1-yl)-2-(2-oxopyrrolidin-1-yl)hexanoates as potential transdermal penetration enhancers” (Bioorg Med Chem. 2010 Jan 1;18(1):73-9. doi: 10.1016/j.bmc.2009.11.020.) and “Investigation of substituted 6-aminohexanoates as skin penetration enhancers” (Bioorg Med Chem. 2012 Jan 1;20(1):86-95. doi: 10.1016/j.bmc.2011.11.033.) because the authors used these references to describe the mechanism of interaction of the lipids with the skin lipids for penetration across the stratum corneum by the fluidization of the lipid bilayer after treating with these pyrrolidinium lipids. The relevant fragments of the text are the following:

‘SC is attributed to its multilayered wall-like structure, in which terminally differentiated keratinocytes are embedded in an intercellular lipid-rich matrix. Various approaches have been developed in recent decades to overcome the skin barrier properties including physical approaches using iontophoresis, sonophoresis and microneedles, chemical approaches using penetration enhancers, and biochemical approaches using liposomal vesicles and enzyme inhibition’

‘Interestingly, many Azone®-related analogs (including those containing modifications in the lipophilic chain and/or heterocyclic moiety) have been synthesized and examined as potential chemical penetration enhancer’

2. The Materials and Methods section. There is overlap with text from the article “The influence of the structural orientation of amide linkers on the serum compatibility and lung transfection properties of cationic amphiphiles” (Biomaterials. 2011 Aug;32(22):5231-40. doi: 10.1016/j.biomaterials.2011.03.059.) by our group, this is due to the fact that the studies, while independent, employed similar methodology.

‘Pyrrolidine (187mg, 2.64mmol) and K2CO3 (365 mg, 2.62 mmol) were added to the above solution. Reaction was stirred at 80°C for 24 h. The reaction mixture was diluted with 100 mL of ethyl acetate, and washed with water (2 x 100 mL). The organic layer was separated, dried over anhydrous sodium sulfate, and concentrated by rotary evaporator. The residue, upon column chromatographic purification over 60-120 mesh size silica gel column using 4% methanol/chloroform (v/v) as eluent, afforded the pure title’

‘Reaction was stirred at 80°C for 96 h. The reaction mixture was diluted with 100 mL of ethyl acetate, and washed with water (2 x 100 mL). The organic layer was separated, dried over anhydrous sodium sulfate, and concentrated by rotary evaporator. The residue, upon column chromatographic purification over 60-120 mesh size silica gel column using 7% methanol/chloroform (v/v) as eluent, afforded the pure title’

‘Reaction was stirred at 80°C for 24 h. The reaction mixture was diluted with 100 mL of ethyl acetate, and washed with water (2 x 100 mL). The organic layer was separated, dried over anhydrous sodium sulfate, and concentrated by rotary evaporator. The residue, upon column chromatographic purification over 60-120 mesh size silica gel column using 4% methanol/chloroform (v/v) as eluent, afforded the pure title’

3. The Discussion section. There is overlap in the text with articles such as “Chemical Penetration Enhancers for Transdermal Drug Delivery Systems” (Tropical Journal of Pharmaceutical Research, April 2009; 8 (2): 173-179) because this article was used as a reference to illustrate the mechanism of percutaneous penetration of our synthesized lipid. The authors cited verbatim the text from this review paper because it has an important relevance to our study and because the text explained in detail the effect of the pyrrolidine structure in our lipids on skin permeation. We should however have cited this in quotation and we strongly apologize for that.

‘Pyrrolidones have been used as permeation enhancers for numerous molecules including hydrophilic (e.g. mannitol and 5-flurouracil) and lipophilic (progesterone and hydrocortisone) permeants.’

‘employed with limited success as a penetration enhancer for captopril formulated into a matrix-type transdermal patch. The pyrrolidones partition well into human stratum corneum within the skin tissue and they may act by altering the solvent nature of the membrane’

‘Such a reservoir effect offers a potential for sustained release of a permeant from the stratum corneum over extended time periods.’

‘percutaneous drug absorption has been increased by a wide variety of long-chain fatty acids, the most popular of which is oleic acid. It is of interest to note that many penetration enhancers such as azone contain both saturated or unsaturated hydrocarbon chains and some structure - activity relationships have been drawn from the extensive studies of Aungst who employed a range of fatty acids, acids, alcohols, sulphoxides, surfactants and amides as enhancers for naloxone’

The identified issues have no bearing on the results and conclusions of the study. The present study, together with its results and conclusion, is new and has not been deduced from these other articles referenced in the article. The authors apologize for the instances of plagiarism noted above.

## References

[1] Marepally S, Boakye CHA, Shah PP, Etukala JR, Vemuri A, et al (2013) Design, Synthesis of Novel Lipids as Chemical Permeation Enhancers and Development of Nanoparticle System for Transdermal Drug Delivery. PLoS ONE 8(12): e82581 doi:10.1371/journal.pone.0082581 24349315 10.1371/journal.pone.0082581 PMC3861410

